# Modeling the catarrhal stage of *Bordetella pertussis* upper respiratory tract infections in mice

**DOI:** 10.1242/dmm.049266

**Published:** 2022-05-03

**Authors:** Illiassou H. Soumana, Kalyan K. Dewan, Bodo Linz, Israel Rivera, Longhuan Ma, Laura K. Howard, Amanda D. Caulfield, Colleen J. Sedney, Uriel Blas-Machado, Peter Sebo, Eric T. Harvill

**Affiliations:** 1 Department of Infectious Diseases, College of Veterinary Medicine, University of Georgia, Athens, GA 30602, USA; 2 Department of Pathology, Athens Veterinary Diagnostic Laboratory, College of Veterinary Medicine, University of Georgia, Athens, GA 30602, USA; 3 Laboratory of Molecular Biology of Bacterial Pathogens, Institute of Microbiology of the ASCR, Czech Academy of Sciences, 14220 Prague 4, Czech Republic

**Keywords:** *Bordetella pertussis*, Catarrhal stage, Shedding, TLR4 receptor, Mouse

## Abstract

Pertussis (whooping cough) is a highly transmissible human respiratory disease caused by *Bordetella pertussi*s, a human-restricted pathogen. Animal models generally involve pneumonic infections induced by depositing large numbers of bacteria in the lungs of mice. These models have informed us about the molecular pathogenesis of pertussis and guided development of vaccines that successfully protect against severe disease. However, they bypass the catarrhal stage of the disease, when bacteria first colonize and initially grow in the upper respiratory tract. This is a critical and highly transmissible stage of the infection that current vaccines do not prevent. Here, we demonstrate a model system in which *B. pertussis* robustly and persistently infects the nasopharynx of TLR4-deficient mice, inducing localized inflammation, neutrophil recruitment and mucus production as well as persistent shedding and occasional transmission to cage mates. This novel experimental system will allow the study of the contributions of bacterial factors to colonization of and shedding from the nasopharynx, as occurs during the catarrhal stage of pertussis, and interventions that might better control the ongoing circulation of pertussis.

## Abstract (precis)

**Summary:** Mouse model of the highly infectious catarrhal stage of pertussis that restricts *Bordetella pertussis* infection to the upper respiratory tract and exhibits nasopharyngeal inflammation and high levels of shedding.

## INTRODUCTION

Pertussis (whooping cough) is caused by the Gram-negative bacterium *Bordetella pertussis*, a highly contagious human respiratory pathogen that was among the leading causes of infant mortality before the introduction of efficacious vaccines (Melvin et al., 2014). The overt symptoms of disease that gradually develop over time include intense fits of spasmodic coughing with the struggle to inhale air (Melvin et al., 2014; https://www.cdc.gov/pertussis/clinical/features.html). Vaccines against pertussis have understandably focused on preventing these severe symptoms and have been developed using animal models of fulminant disease induced by inoculating large doses of the pathogen deep into their lungs. Current acellular vaccines confer protection against symptoms of severe disease but do not prevent nasopharyngeal infections that induce non-specific cold/flu-like symptoms that allow the spread of infection (Warfel et al., 2014). Evidence that transmission from such asymptomatic infections is among the factors driving the worrisome resurgence of pertussis in countries that use these vaccines (van der Ark, et al., 2012; Witt et al., 2013; Craig et al., 2020; Gill et al., 2021) has led to efforts to strengthen protection in the nasopharynx to prevent colonization and transmission (Allen et al., 2018; Holubová et al., 2020; Wolf et al., 2021).

With most research on pertussis being focused on the overt symptoms of severe disease modeled in pneumonic infections in mice, much less is known about the early stages of *B. pertussis* colonization and growth within the nasopharynx, which that model does not mimic. Clinically, typical symptoms in the majority of adult human infections develop sequentially in three distinct phases (https://www.cdc.gov/pertussis/clinical/features.html). The first stage is the catarrhal stage, encompassing the early incubation phase of nasopharyngeal infection which may last ∼2 weeks. Infected people exhibit any of a range of non-specific symptoms often mistaken for a mild cold/flu, such as low-grade fever, malaise or sore throat, nasal congestion, rhinorrhea, lacrimation, sneezing and mild progressive dry cough (Kilgore et al., 2016). This period is then followed by the paroxysmal stage, in which coughing symptoms intensify over ∼4-6 weeks, and finally a prolonged convalescent stage, in which symptoms gradually diminish over several weeks. The paroxysmal stage, with overt symptoms involving recurring bouts of intense coughing, was quite rapidly recognized to be contagious, with the cough-plate being used to capture aerosolized bacteria as a diagnostic method (Kendrick and Eldering, 1934). More recent experiments using baboons with experimentally established *B. pertussis* infections that induce coughing, have also demonstrated that infected animals can transmit (Warfel et al., 2014). The same study on baboons also showed that animals vaccinated with the acellular pertussis vaccine (aPV), although protected from severe disease, still harbored mild infections of the nasopharynx and could transmit to other animals. In humans, similarly mild infections are not recognized clinically, making chains of transmission difficult to track. Longitudinal surveillance of subclinical symptoms and healthy contacts yielded evidence of undiagnosed human infections contributing to transmission (Gill et al., 2021), indicating that paroxysmal cough is not necessary for efficient transmission. However, despite the catarrhal stage being considered a highly contagious stage of the infection for humans (Hoey, 2003; Tozzi et al., 2005; Lauria and Zabbo, 2020) there are no established animal models of the catarrhal stage of infection.

Our recent exploration of very low dose inoculation of the nasal cavity provided strong evidence that *B. pertussis* interactions with the host are very different in the upper respiratory tract than in the lungs (Soumana et al., 2021). To further explore the catarrhal stage of infection, we set out to induce more severe disease that is limited to the upper respiratory tract, predicting that better understanding this stage will contribute fresh perspective to guide the development of interventions to prevent colonization, shedding and spread of the pathogen.

Initial host-pathogen interactions are mediated via pathogen-associated molecular pattern recognition by host toll-like receptors (Kawasaki and Kawai, 2014), such as the critical recognition of *B. pertussis* lipopolysaccharide (LPS) by TLR4. This becomes relevant when using the mouse as a model because the penta-acylated lipo-oligosaccharides of *B. pertussis* are less stimulatory to human than to mouse TLR4-MD-2 receptor complexes, when compared to the hexa-acylated LPS of its apparent progenitor, *Bordetella bronchiseptica* (Hajjar et al., 2002*)*. In its adaptation to become a highly infectious human pathogen, *B. pertussis* (and *Bordetella parapertussis*) appears to have modified its LPS to be much less stimulatory by diminishing TRL4-mediated inflammatory signals, thereby facilitating human colonization (Higgins et al., 2003; Mann et al., 2005). But robust recognition by mouse TLR4 contributes to the rapid control of *B. pertussis* infections in the upper respiratory tracts of mice (Mann et al., 2005; Wolfe et al., 2009), preventing the efficient study of the early and prolonged catarrhal stage of upper respiratory infection.

To explore the possibility that TLR4 signaling might interfere with growth, shedding and transmission in mice, we employed C3H/HeJ mice that are defective in TLR4 signaling. We previously reported that the topical administration of antibiotics to the nasal cavities of mice allowed low numbers of *B. pertussis* to successfully colonize and grow there (Weyrich et al., 2014). When delivered intranasally in low volumes and low numbers, colonization was restricted to the nasal cavity, was more prolonged and induced no overt symptoms to indicate infection. (Soumana et al., 2021). Based on these earlier observations, here we delivered *B. pertussis* to similarly antibiotic-treated mice, in low volumes to limit bacterial colonization to the nasopharynx, but used large numbers of colony forming units (CFUs) to ensure robust colonization restricted largely to the nasopharynx. When inoculated in this way, *B. pertussis* induced several of the key features of the catarrhal stage of human infection, including inflammation, mucus secretion, as well as early and sustained profuse shedding. Importantly, these symptoms arose without the induction of the strong systemic immune response observed in the conventional pneumonic inoculation model, highlighting the very different immune responses to lung versus nasopharyngeal infections. We propose that this nasopharyngeal infection model can be complementary to the conventional pneumonic model and may be more appropriate for the study of the catarrhal stage of upper respiratory infection. In addition, this model may be well suited for identification of candidate vaccine antigens that can potentially block colonization and shedding of *B. pertussis* from the upper respiratory tract.

## RESULTS

### *B. pertussis* efficiently colonizes the nasopharynx

We have previously reported the successful infection of mice using low numbers of *B. pertussis* (<500 CFU) delivered in low volumes (5 µl PBS) to mice nasally pretreated with antibiotics to remove/perturb resident nasal microbiota (Weyrich et al., 2014). Delivered this way, the bacteria grew in numbers and remained localized to the nasal cavity, inducing no significant symptoms (Soumana et al., 2021). Based on these earlier observations, here we also delivered *B. pertussis* to antibiotic-treated mice, using low volumes but delivering larger numbers of bacteria to induce a robust host response limited to the nasopharynx.

**Fig. 1.**
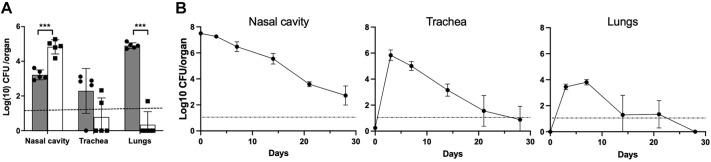
**Distribution of *B. pertussis* across respiratory organs following inoculation via the conventional high volume ‘pneumonic’ inoculation versus a low volume ‘nasopharyngeal’ inoculation.** (A) The graph shows the distribution of *B. pertussis* in the respiratory tracts of mice 2 h after being inoculated with 50 µl (pneumonic, gray columns) or 5 µl (nasopharyngeal, white columns). *n*=5 mice per group. Data are mean±s.e.m. ***P<0.001 (two-way ANOVA). Dotted line represents the limit of detection. Experiment was conducted once. (B) The colonization profile of the catarrhal model, with graphs depicting the colonization loads (CFU) in respiratory organs (nasal cavity, trachea and lungs) of mice over time following inoculation with 2.5×10^7^ CFU *B. pertussis* in 5 µl PBS (‘catarrhal’ model). Filled circles represent the mean±s.d. (*n*=4). Dotted line represents the limit of detection. Experiment was conducted twice.

To examine how higher CFU inocula would distribute across the respiratory tract when using the conventional approach versus our low-volume delivery approach, we inoculated groups of C3H/HeJ mice (*n*=5 per group) with the conventionally used dose (5×10^5^ CFU) delivered in either 5 or 50 µl of PBS. Fig. 1A shows that 2 h after inoculation in high volume (50 µl), the majority of the inoculum was recovered from the lungs. In contrast, when bacteria were delivered in a low volume (5 µl) *B. pertussis* was overwhelmingly (>99.99%) localized to the nasal cavities and remained undetected in trachea and lungs of most animals. These results show that controlling delivery volumes can allow colonization to be localized exclusively to the upper respiratory tract, providing an opportunity to study the potentially different immune interactions in the upper respiratory tract from the lower respiratory tract.

To observe how infections localized to the nasopharynx progress, spread and persist over time, groups of C3H/HeJ mice were inoculated with higher numbers of *B. pertussis* (∼2.5×10^7^ CFU) in 5 µl PBS (Fig. 1B). Three days later, *B. pertussis* was recovered in large numbers (∼10^7^ CFU) from the nasal cavity and had spread to the lungs, where they grew to reach close to 10^4^ CFU within a week. Interestingly, bacterial numbers in the lower respiratory tract did not increase further but declined sharply and were near or below detection limits (10 CFU) by day 14. This decline inversely correlated with an increase in serum IgG titers (Fig. S1) (Kirimanjeswara et al., 2003), indicating that the host immune response protects the lungs from severe infection, consistent with the notion that, in adults, pertussis is essentially a disease of the upper respiratory tract (Cherry, 2013, 2015). Unlike in the lungs, numbers of bacteria in the nasal cavity and trachea decreased much more slowly, indicating substantially different immune-mediated clearance from the lower and upper respiratory tract. These data strongly support the rationale for the use of this experimental system to study upper respiratory infection, representing the catarrhal stage, as different in important regards from the conventional study of lower respiratory (pneumonic) infection.

### Histopathological markers for the catarrhal stage of infection

**Fig. 2.**
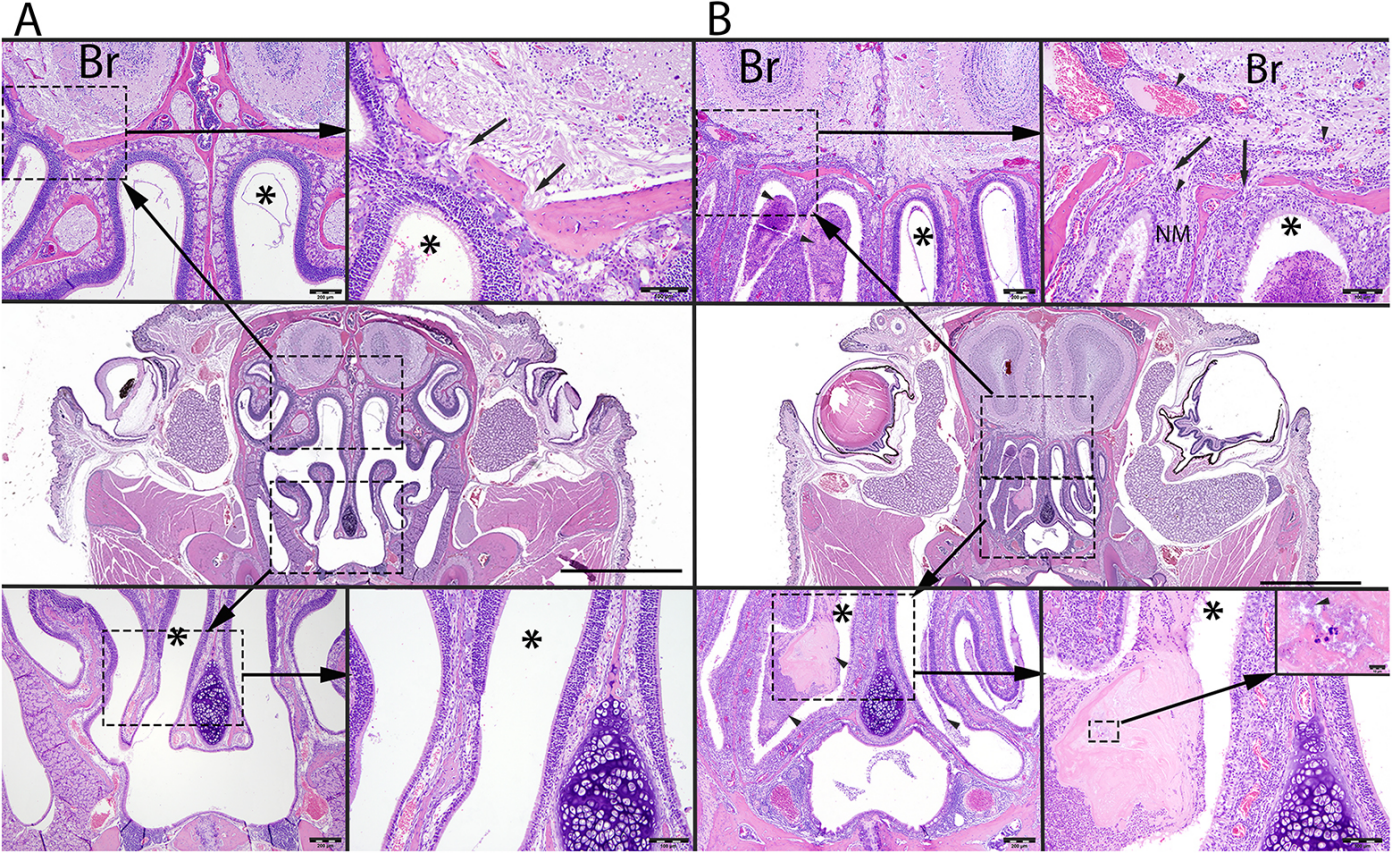
***B. pertussis*-induced histopathology of mouse nasal cavity.** (A,B) Representative images from control (A) and *B. pertussis*-challenged (B) mice obtained from Hematoxylin and Eosin (HE)-stained whole-slide scanned images of coronal section of the nose at the level of the eyes and olfactory bulb of the brain (Br). Scale bars: 2 mm (middle panels). Compared to controls (A), mice inoculated with *B. pertussis* (B) have large numbers of neutrophils, mixed with mucus, proteinaceous material and bacteria filling the nasal meatus (asterisks). In A, the top left image illustrates the dorsal nasal meatus (asterisk) and its close association to the olfactory bulb of the brain (Br). Scale bar: 200 µm. The top right image illustrates a higher magnification of the cribriform plate (arrows) between the brain (Br) and the nasal meatus (asterisk). Scale bar: 100 µm. The bottom left image illustrates the mid- and ventral nasal meatus (asterisk). Scale bar: 200 µm. The bottom right image illustrates a higher magnification of the mid-nasal meatus (asterisk). Scale bar: 100 µm. In B, the top left image illustrates a mucopurulent exudate (arrowheads) within the dorsal nasal meatus (asterisk). Scale bar: 200 µm. The top right image illustrates a higher magnification of the cribriform plate (arrows) between the brain (Br) from the nasal meatus (asterisk). Increased number of inflammatory cellular infiltrates (arrowheads) obscure the nasal mucosa (NM) and extend along the perivascular spaces through the cribriform plate into the olfactory bulb of the brain (Br). Scale bar: 100 µm. The bottom left image illustrates the mid- and ventral nasal meatus (asterisk) filled with mucopurulent exudate (arrowheads). Scale bar: 200 µm. The bottom right image illustrates a higher magnification of the mid-nasal meatus (asterisk) containing mucopurulent exudate. Scale bar: 100 µm. The insert at the top represents a higher magnification of mucopurulent exudate mixed with bacteria (arrowhead). Scale bar: 10 µm. See Fig. S2 for additional details.

To investigate the tissue damage associated with robust nasopharyngeal infections, mice were inoculated intranasally with *B. pertussis* as above, or with PBS as control, in 5 µl volumes. Histopathological evaluations (Table S2) were made on Hematoxylin and Eosin (H&E)-stained coronal sections through the nose on day 7 post inoculation. Fig. 2 shows representative images from uninfected control (left panel) and infected (right panel) mice. Compared to uninfected controls, inoculated mice had significant nasal inflammation (rhinitis), which extended throughout the length of the nose. A layer of mucopurulent exudate covered the focally denuded mucosa of the nasal cavity, which exhibited a loss of cilia, scattered neutrophils, necrotic epithelia and loss of goblet cells. The exudate included large numbers of neutrophils, sloughed epithelial cells, bacteria and necrotic debris embedded in pale basophilic, mucinous material or mucus. Mild to moderate numbers of neutrophils, with fewer lymphocytes and plasma cells, obscured the underlying lamina propria. In some areas, Gram-negative bacterial rods were observed between the cilia mixed with the mucopurulent material. In other areas, the maxillary turbinates were fused, and the supporting bony structures were irregular. At the level of the cribriform plate, neutrophils, with fewer lymphocytes and plasma cells, extended into the adjacent meninges (lining) of the olfactory bulb of the brain, expanded the perivascular tissues of blood-engorged vascular channels, and occasionally extended into the adjacent neuropil (brain tissue). These results indicate that high CFU numbers of *B. pertussis* localized to the nose can induce significant inflammation, epithelial damage, and mucus and exudate production, providing an opportunity to study these aspects of the catarrhal stage of infection (Fig. S2).

### Activation of chitinase-like genes, neutrophil-derived glycoproteins and complement

An RNA-seq gene expression analysis in the nasal cavity of mice infected with *B. bronchiseptica*, a close relative and proposed ancestor species of *B. pertussis* (Diavatopoulos et al., 2005; Linz et al., 2016), revealed induction of a strong innate immune response starting as early as 3 days post-inoculation and lasting throughout the experiment to day 21 (I.R., B.L. and E.T.H, unpublished). A greater than 5-fold upregulation was observed in transcripts of *Chil1*, encoding a chitinase-3-like glycoprotein involved in modulating infection and inflammation (Lee et al., 2011; Di Rosa et al., 2016) and *CD177*, encoding a glycosylphosphatidylinositol-linked cell surface antigen mediating neutrophil proliferation (Sachs et al., 2007; Bayat et al., 2010). In addition, an upregulation of transcripts for *C3* (complement component 3), which plays a central role in complement activation and immunosurveillance (Sahu and Lambris, 2001), and of mucin genes *Muc1*, *Muc4*, *Muc5ac* was also noted (Table S3).

**Fig. 3.**
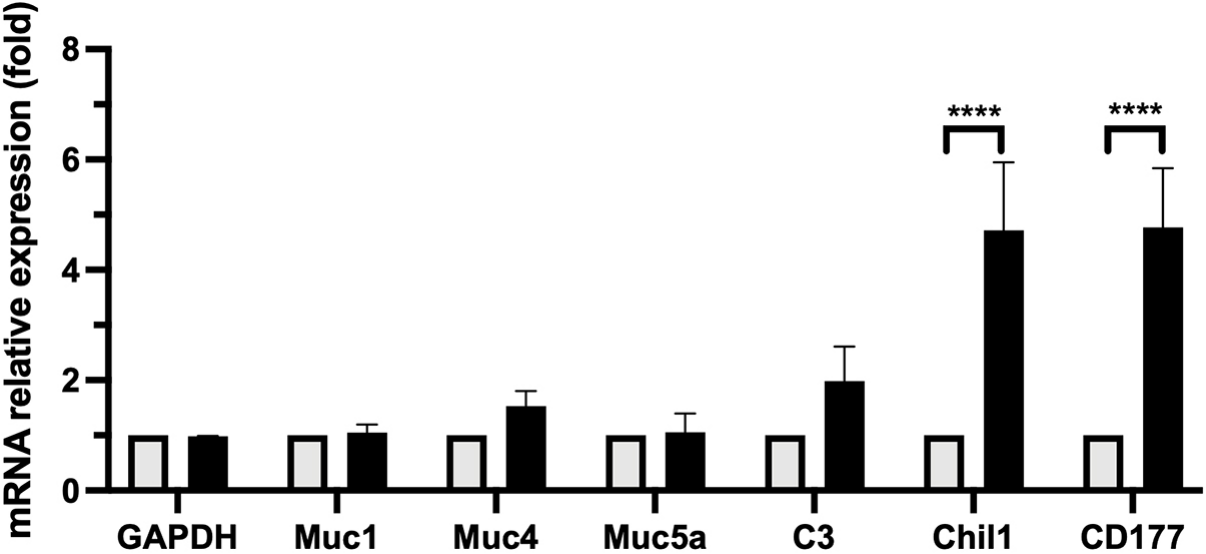
**Upregulation of gene transcripts during the catarrhal stage detected by RT-qPCR.** Graph shows comparative expression levels of genes in naïve uninfected mice (PBS-treated; light gray columns) compared with high dose nasopharyngeal inoculation with *B. pertussis* infection (black columns). Results show strong upregulation of *Chil1* and *CD177* genes compared to PBS-treated mice 3 days post infection. Moderate but not significant upregulation of *C3* and *Muc4* genes were observed. In contrast, expression of *Muc1* and *Muc5ac* was unchanged. *n*=4 mice. ****P<0.0001 (two-way ANOVA). Experiment was conducted twice.

To assess the expression of these genes associated with nasopharyngeal inflammation induced by nasopharyngeally inoculated *B. pertussis*, mRNA was isolated from dissected nasal cavities of naïve (control) and *B. pertussis* challenged mice 3 days post inoculation and the transcript expression levels of the above genes was analyzed using RT-qPCR (Fig. 3). *B. pertussis* infection did not alter transcript levels of *Gapdh* (control), or mucin genes *Muc1* or *Muc5ac*. Infected animals appeared to have somewhat higher transcript levels of *Muc4* and *C3*, although these differences were not statistically significant. As seen in *B. bronchiseptica*, *CD177* transcript levels were elevated nearly 5-fold in the infected group compared to controls, supporting the observed accumulation of neutrophil in the nasal meatus. In addition, transcript levels of *Chil1* were similarly increased 5-fold compared to the uninfected controls, making it another prominent marker of the host immune response during the catarrhal stage.

### Nasopharyngeal inoculation results in prolonged high levels of shedding

**Fig. 4.**
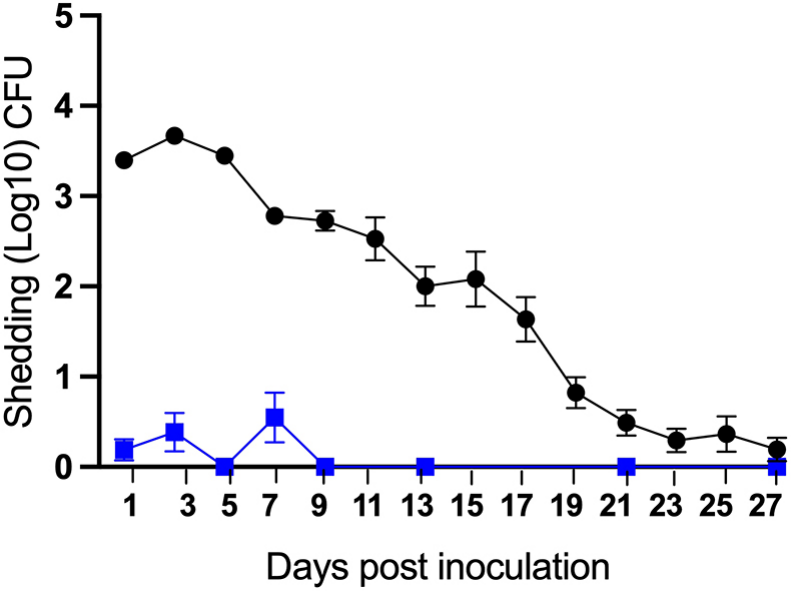
**Shedding profile of catarrhal stage infection.** *B. pertussis* CFUs recovered on indicated days from the nose tips of C3H/HeJ (black circles, *n*=10) and C57BL/6J (blue squares, *n*=5) mice inoculated with 5×10^7^ CFU of *B. pertussis* delivered in 5 μl PBS. Data are mean±s.d. Experiment was conducted twice.

The potential of an infected host to transmit to others depends to a large extent on how much the host sheds the pathogen. To examine potential shedding in this model, we inoculated groups of C3H/HeJ (*n*=10) and C57BL/6J (*n*=5) mice as described earlier and sampled the shedding of *B. pertussis* from the external nares every 2 days. As shown in Fig. 4, C57BL/6J mice shed few, if any, bacteria over most time points, repeating the failure of previous experiments attempting to observe transmission between adult mice. In striking contrast, shedding was observed from all inoculated C3H/HeJ mice at multiple consecutive time points, with thousands of bacteria recovered at each time point for about a week, slowly decreasing and showing greater variability thereafter. The numbers shed at every time point remained above detection limits in all mice until day 17 (Fig. 4). These results demonstrate a critical role for TLR4 signaling in activating innate immune-mediated control of *B. pertussis* numbers within the nose and being shed into the environment to transmit to others*.* They also potentially explain the selective advantage of the lipid A changes that accompany the adaptation of *B. pertussis* to a highly contagious acute infection strategy in humans. Further, these results agree with the notion of pronounced shedding in the early catarrhal stage of human infection, a period when paroxysmal coughing symptoms have not yet developed but when transmission is likely to be driven by rhinorrhea, sneezing and mild cough.

### Transmission in the catarrhal stage infection model

**Fig. 5.**
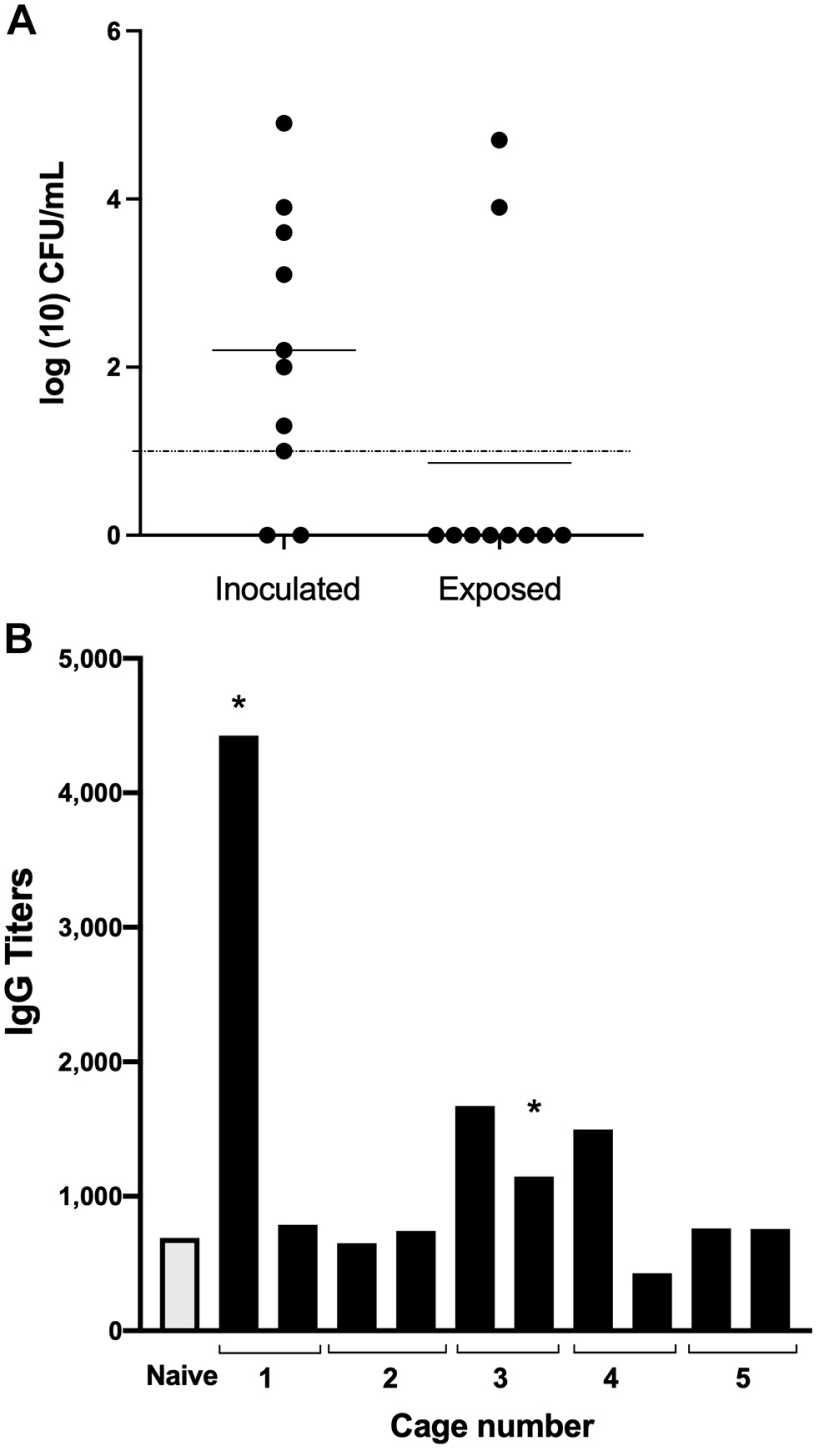
**Transmission of *B. pertussis* among adult mice and seropositivity of anti-*B. pertussis* IgG in exposed mice**. (A) Filled circles on graph represent the number of *B. pertussis* CFUs recovered from the nasal cavities of individual mice that had been inoculated with *B. pertussis* (Inoculated) or co-housed with the inoculated mice (Exposed) for 28 days (two inoculated+two naïve; *n*=5 cages). Horizontal bar indicates the mean. Dashed line indicates the level of detection. Experiment was conducted twice. (B) The graph shows the relative titers of anti-*B. pertussis* IgG antibodies detected in individual sera of 10 exposed mice co-housed in five cages (from A) for 28 days with mice inoculated via catarrhal model (see text for description). Asterisk indicates culture-positive mice identified in A. Experiment was conducted twice.

The extensive shedding following localized nasal inoculation suggested that we might observe transmission between mice under these conditions. To detect transmission between mice, pairs of inoculated mice were co-housed with two uninfected (exposed) mice (*n*=5 cages). After 28 days of co-housing, we detected *B. pertussis* in the nasal cavities of two exposed mice, indicating that transmission can occur in low frequencies under these conditions (Fig. 5A). The relatively high numbers (∼10,000 CFU) recovered from the nasal cavities of the two exposed mice indicate that, once transmitted to exposed animals, *B. pertussis* can colonize and grow efficiently in mice.

As the possibility that some exposed mice had been colonized but had cleared their infection could not be ruled out, ELISA assays were conducted to screen for anti-*B. pertussis* IgG antibodies as evidence of prior transient colonization. The two mice (asterisks) that were shown to be colonized by *B. pertussis* (Fig. 5B) had at least 2-fold higher antibody titers than the naïve control. Two other mice had >2-fold elevated levels of anti-*B. pertussis* antibodies, suggesting other mice may have been transiently colonized with *B. pertussis* (Fig. 5B). We obtained very similar results when we repeated the cohousing transmission experiment (Fig. S3), with 2 out of 10 naïve co-housed mice becoming colonized. Among the 10 co-housed naïve mice, we again detected four mice with elevated serum IgG titers, which included the two mice that were colonized. These observations confirm that transmission takes place under these conditions and again suggest that transient infections may have occurred among the exposed mice.

## DISCUSSION

During the catarrhal stage of nasopharyngeal *B. pertussis* infection, the rate of transmission may be less dependent on severe paroxysmal coughing than on the amounts of pathogen being shed via relatively non-specific cold/flu-like symptoms. In addition, experimental work with aPV-vaccinated baboons has shown that aPV vaccination prevents the most severe coughing symptoms but does not prevent *B. pertussis* from colonizing the nasopharynx and transmitting to other animals. In this respect, infections in both vaccinated and unvaccinated hosts appear to be in a transmission-competent state. As noted previously with *B. bronchiseptica* (Rolin et al., 2014), TLR4-deficient (C3H/HeJ) mice shed consistently higher numbers of bacteria*,* indicating that TLR4 interferes with shedding during this period. It is noteworthy that the most robust shedding we observed occurred during the first 5 days of infection and decreased thereafter, indicating that *B. pertussis* is likely to be highly contagious during the early days of nasopharyngeal infection, as is considered for humans during the catarrhal stage (Hoey, 2003; Tozzi et al., 2005). It would be reasonable to infer that the striking lack of shedding in C57BL/6J mice compared to the C3H/HeJ mice (Fig. 4) reflects profound differences in the interactions that the *B. pertussis* LPS has with murine TLR4 receptors (Schroder et al., 2012; Vaure and Liu, 2014). Penta-acylated lipo-oligosaccharides, which are found in human adapted *B. pertussis*, are less stimulatory towards human TLR4 when compared to the hexa-acylated lipo-oligosaccharides found in *B. bronchiseptica* (Hajjar et al., 2002). If a decreased TLR4 response during early *B. pertussis* infection in humans, relative to that of mice, is crucial for high levels of colonization, shedding and transmission, then it is likely that these features that we observe in C3H/HeJ mice harboring a compromised TLR4 receptor would recapitulate this interaction. This would also explain why *B. pertussis* transmission has never been observed among adult immunocompetent mice. In any case, the ability of *B. pertussis* to modulate the human TLR4 response via its altered LPS provides a strong rational for the use of the C3H/HeJ model to mimic these effects. It is only possible to study aspects of interactions that we can observe and measure, and this experimental system might provide an important new way to examine and study bacterial factors that contribute to inducing shedding, survival between hosts, colonization of new hosts and other mechanistic aspects of the transmission process that cannot effectively be studied in humans or baboons.

The transcript for *Chil1*, a gene encoding a protein that is known to modulate the anti-inflammatory response to pathogens (Lee et al., 2011), was significantly upregulated by *B. pertussis*. But the nasal cavities did exhibit inflammation, with a large influx of neutrophils and the accumulation of mucus and proteinaceous granules in the nasal meatus; features that are likely to contribute to efficient shedding. Inflammatory conditions were also observed in high-dose aerosol challenged C3H/HeJ mice (Higgins et al., 2003), indicating that TLR4-independent routes to inflammation induced by *B. pertussis* mediate efficient shedding. *C3*, a key mediator of complement activation and a potent immunosurveillance protein (Sahu and Lambris, 2001; Ricklin et al., 2016), was also upregulated, likely contributing to inflammation. In spite of the histological evidence of mucus secretion being elevated, we found no corresponding increase in the transcript expression levels of the mucin genes (*Muc1*, *Muc4*, *Muc5ac*) at the time point examined (day 3). However, the complexities in the dynamics of mucin synthesis, storage and exocytosis in respiratory airways (Adler et al., 2013) makes it difficult to link transcript upregulation at this specific time point to mucus that had already accumulated by then.

Transmission has previously been observed between newborn (7 to 14 day old) mice (Scanlon et al., 2017), but the difficulty of working with these mice, including their high susceptibility to lethal pneumonia, substantially different immune system and very narrow time window likely make this model most useful as a model of the extreme case of *B. pertussis* in neonatal humans. As a complementary approach, the experimental system presented here provides the first report of the transmission of *B. pertussis* between adult mice. Transmission was modestly efficient, with small proportions of exposed mice confirmed colonized with *B. pertussis* in this 28-day period*.* Other exposed mice had elevated anti-pertussis IgG titers, suggesting that they may have been transiently colonized, but this remains a minority of the animals exposed. This initial observation of *B. pertussis* transmission among adult mice may allow further optimization, for example by using even more transmission-prone mice, altering the conditions and length of exposure, developing non-lethal modes of analysis, increasing the number of mice infected or changing the ratios of inoculated and exposed mice. We are currently exploring a derivative model lacking TLR signaling via its lack of MyD88 (MyD88^−/−^), which appears to further increase transmission. With increased time of exposure, the detection of anti-*B. pertussis* antibodies may more sensitively report on nasal colonization and/or allow the study of animals that successfully repel colonization.

Clinical, epidemiological and experimental observations indicate that the current resurgence of pertussis is being driven by patients who can be asymptomatic or show an atypical suite of symptoms that are much milder and cold/flu-like. This may be because of the short duration of protection conferred by acellular vaccines or their inability to prevent nasopharyngeal infections (Holubová et al., 2020; Dubois et al., 2021). Whatever the reasons, improvements in vaccines to prevent nasopharyngeal infections and transmission are a pressing need, and assays of nasopharyngeal colonization, growth, shedding and transmission are required for their development.

Although the presented experimental model may not be appropriate to address all questions, it provides a first approach to study the nasopharyngeal-localized pathological characteristics of the early stage of *B. pertussis* infection, including the shedding and transmission between animals. In addition to its likely utility in the development of vaccines that block nasal colonization, pathogenesis and shedding, this experimental system may be useful to determine the roles of many *B. pertussis* factors during this important stage of infection, for which efforts are already underway (Holubova et al., 2022). It is worth noting that nearly all known *B. pertussis* factors have been studied using inoculation methods that deliver bacteria into the lungs (pneumonic infection model). Indeed, factors that affect the outcomes of this model are generally referred to as ‘virulence factors’, based on their contribution to virulence as measured in this model of extreme, near lethal pneumonic disease. The nasopharyngeal-localized infection observed here is likely to provide different and valuable windows through which to observe the effects of *B. pertussis* factors within the upper respiratory tract. We, and others, have demonstrated that the immune response within the upper respiratory tract is substantially different from that in the lower, and this system may allow interactions between *B. pertussis* and the former to be probed independently from the latter.

We readily embrace the truism, attributed to George Box: “All models are wrong. Some models are useful.” All models are approximations; the question is whether they can provide useful information that can inform our understanding and guide our development of improved treatments and preventatives. Considering the relative failure of conventional pneumonic infection models to predict the limited effect of current vaccines on upper respiratory tract infections and transmission, a complementary model that more accurately mimics those may be highly useful in the design of the next generation of vaccines.

## MATERIALS AND METHODS

### Bacterial culture and mouse infections

*B. pertussis* strain 536 (Harvill et al., 1999; Parkhill et al., 2003) was maintained on Bordet-Gengou (BG) agar (Becton Dickson, 248200) with 10% sheep blood (HemoStat, 644000-1) and 20 μg/ml streptomycin (Acros Organics, 455341000), incubated for 5 days at 37°C and grown in Stainer-Scholte broth (Stainer and Scholte, 1970) at 37°C with shaking at 200 rpm (VWR, Advanced 3500 Orbital Shaker). Antibiotic pretreatment involved gently pipetting 10 μl of PBS (pH 7.4) containing 45 μg of gentamicin (GentaFuse™, Henry Schein, 006913) delivered in 10 μl of PBS on three separate occasions 12 h apart onto the external nares of mice anesthetized with 5% isoflurane (Pivetal, P151A) with the final treatment 12 h before inoculation. For nasopharyngeal inoculations (catarrhal model), *B. pertussis* was delivered by pipetting 5 µl PBS containing 3-4×10^7^ CFU onto the external nares of anesthetized mice. For the deep lung inoculations (pneumonic model), 50 µl PBS containing 5×10^5^ CFU of bacteria was used. To assess the colonization load of respiratory organs, mice were euthanized by CO_2_ inhalation (1.5 l/min) and the dorsal bones and internasal septum and soft tissue of nasal cavities, lungs and tracheas were excised, disrupted for 45 s using 2.8 mm ceramic beads (Omni International, 19-646-3) on a bead homogenizer (Fischerbrand, Bead Mill 24), serially diluted in 1 ml PBS and 100 µl of each dilution plated on BG agar (limit of detection 10 CFU).

### Bacterial shedding

Shedding of *B. pertussis* was quantified by tapping the mouse nose five times on the surface of a BG agar plate and 100 μl of PBS was then used to uniformly spread the deposited bacteria before culture and enumeration.

### Transmission

Inoculated mice (index mice) were tagged to facilitate their identification and cohoused with uninfected (exposed) mice. On day 28, the respiratory tract organs were assessed as above.

### Histology

Five-week-old female C3H/HeJ or C57BL/6J mice were assessed (*n*=6) on day 7 post infection. Following fixation in neutral-buffered 10% formalin solution (StatLab, 28600-5) and subsequent decalcification in Kristensen's solution, coronal sections were made through the nose and brain, and transverse sections were made through the middle and inner ear. Tissues were embedded in paraffin, sectioned at ∼5 µm, and stained with H&E. Histopathological examination consisted of evaluation of the nose for the incidence (presence or absence), severity and distribution of inflammation.

### RNA extraction and RT-qPCR analysis

Nasal cavity tissues were stored in 1 ml PBS at −80°C until extraction using the PureLinkTM RNA Mini kit (Ambion, 12183025) following the manufacturer's instructions. cDNA was generated from each sample using a high-capacity reverse transcription kit (Applied Biosystems). Approximately 10 ng cDNA was used as a template in reactions with 0.5 µM each forward and reverse primers (Table S1) and SYBR Green (Applied Biosystems, Power SYBR Green Mastermix, 4367659) according to the manufacturer's instructions. Reactions were carried out using the Quant Studio 5 (Applied Biosystems), with 5 min pre-incubation at 95°C, followed by 40 cycles of a two-step PCR at 95°C and 60°C and fold changes were calculated using the ΔΔCT method. Expression of the *Gapdh* gene was used as an endogenous control.

### ELISA assay

Assays were performed according to a previously published protocol from our lab (Hester et al., 2013). Briefly, 96-well Nunc microtiter plates (Thermo Fisher Scientific, 80040LE 0910) were coated with heat-killed *B. pertussis* and incubated in a humidified chamber at 35°C for 4 h, then blocked with phosphate-buffered saline with 0.1% Tween-20 (PBS-T) and 1% bovine serum albumin and left overnight at 4°C. Reactions were developed with SureBlue™ (Sera Care, 5120 0076) and terminated with 1 M HCL after ten minutes. Color intensities were determined at an optical density (OD) of 450 nm. The titer was determined to be the reciprocal of the lowest dilution in which an OD of 0.1 was obtained.

### Statistical analysis

Data generated were statistically evaluated by a two-tailed unpaired Student's *t*-test and two-way ANOVA using the statistical analyses package of GraphPad prism (V2.0).

### Ethics

Animal housing and all experiments were approved by the Institutional Animal Care and Use Committee of the University of Georgia (*Bordetella* host-pathogen interaction: A2019-03-003-Y2-A8). C3H/HeJ mice were originally obtained from The Jackson Laboratory and maintained under specific pathogen-free conditions at the University of Georgia.

## Supplementary Material

Supplementary information
