# Supplementary material for: Modeling the catarrhal stage of Bordetella pertussis upper respiratory tract infections in mice
Source: Dis Model Mech. 2022 May 3;15(5):dmm049266. doi: 10.1242/dmm.049266 (PMC9092653; doi:10.1242/dmm.049266)
Supplement: Supplementary information [file dmm-15-049266-s1.pdf]

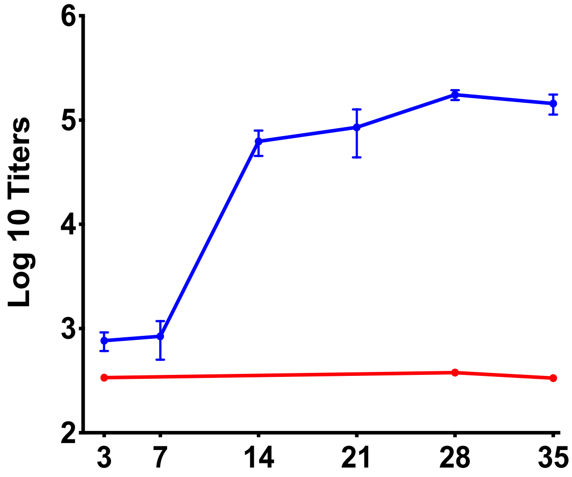

Fig. S1. Average serum anti-*B. pertussis* IgG titers from groups of C3H/HeJ mice inoculated intranasally with a high dose (10^7 CFU/5µL PBS) of *B. pertussis* (blue line) or left uninfected (red line). (n=4 per group)

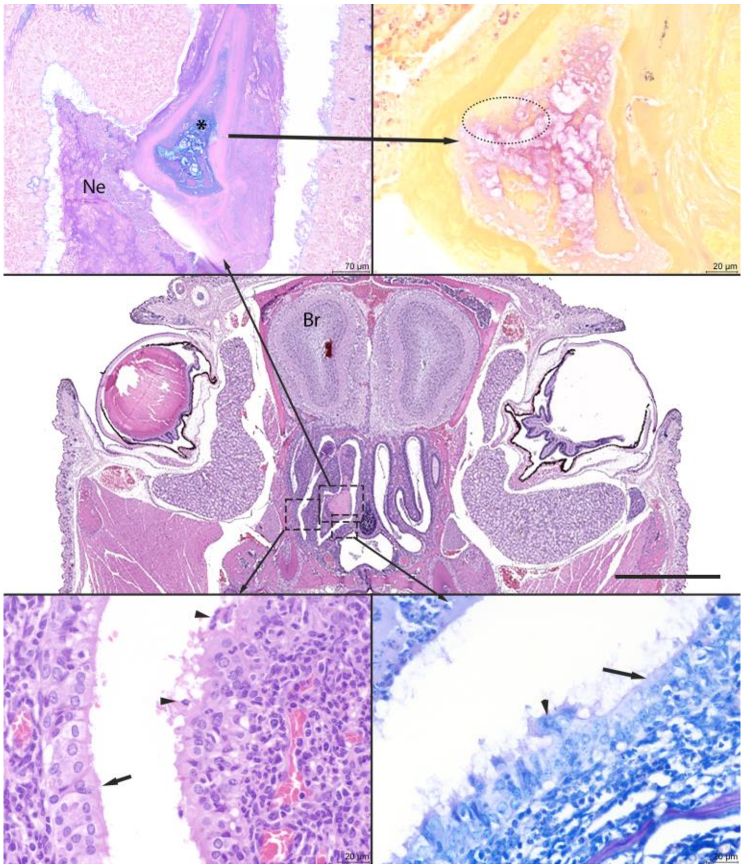

Fig. S2. Histopathology of *B. pertussis* day 7 post infection
Center panel: Representative images from the *B. pertussis* challenged mice obtained from stained whole-slide scanned images of coronal section of the nose at the level of the eyes and olfactory bulb of the brain (Br). HE stain. Scale bar = 2 mm. Dashed rectangles and arrows illustrate higher magnification of affected areas. ***Top left:*** image illustrates a mucopurulent exudate within the nasal meatus (asterisk, *). The exudate has a mixture of neutrophils (Ne) arranged about proteinaceous (pink/purple) and mucous material (blue) in the center. Periodic Acid Schiff-Alcian Blue stain. Scale bar = 70 µm. ***Top right:*** image, the dashed ellipse illustrates a cluster or colony of gram-negative bacteria within the mucous-rich exudate. ***Bottom left***: the long arrow points to ciliated epithelia, and the arrowhead points to sloughed cells lining the nasal turbinate. HE stain. Scale bar = 20 µm. ***Bottom right***: the long arrow points to epithelia with loss of cilia, and the arrow head points to sloughed ciliated cells lining the nasal turbinate. Giemsa stain. Scale bar = 20 µm.

Disease Models & Mechanisms • Supplementary information

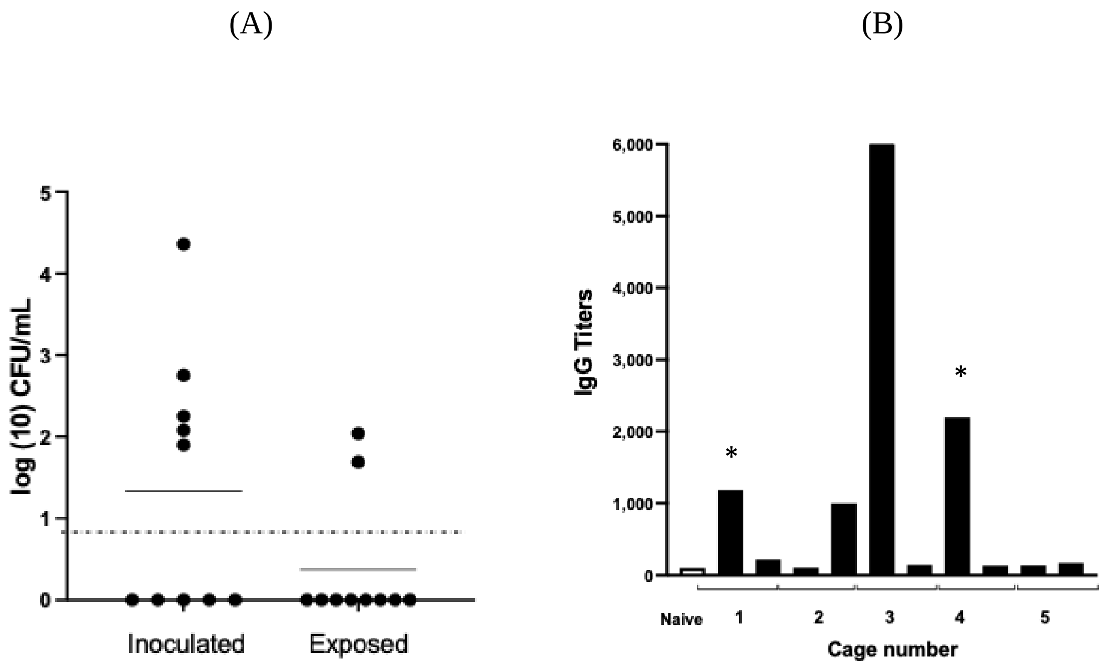

Fig. S3. Transmission of *B. pertussis* among adult mice. **A**.) Filled circles on graph represent the number of *B. pertussis* CFUs recovered from the nasal cavities of individual mice that had been inoculated with *B. pertussis* ("Inoculated") or co-housed with inoculated mice ("Exposed") for 28 days (2 inoculated + 2 naïve; n = 5 cages). The horizontal bar indicates the mean, the dashed line indicates the level of detection. **B.**) Seropositivity of anti *B. pertussis* IgG in exposed mice. The graph shows the relative titers of anti- *B. pertussis* IgG antibodies detected in individual sera of 10 exposed mice co-housed in 5 cages (2 mice per cage) for 28 days with mice inoculated via catarrhal model.

Disease Models & Mechanisms • Supplementary information

Table S1. Primers (Integrated DNA Technologies, United States), used in study to evaluate catarrhal stage expression of targeted genes.

1. Glyceraldehyde-3-phosphate dehydrogenase
   GAPDH Forward: 5’-TCACCATGGAGAAGGC-3’
   GAPDH Reverse: 5’-GCTAAGTTGGTGGTGCA-3’
2. Mucin
   Muc1 F: 5’-GATCTCTAGCATCAAGTTC-3’
   Muc1 R: 5’-CTCATTCACTTTTGACTTCT-3
   Muc4 F: 5’-GATACTCCAAGTTCCATAAC-3’
   Muc4 R:5’-CAGGAGTTGTTCTTTG-3’
   Muc5a F: 5’-CTATCAAGGCTGCTTATTT-3’
   Muc5a R: 5’-GCTGGTATACTTGGTTATC-3’
3. Complement C3
   C3 F: 5’-AGGAATTCAACTCAGATAAG-3’
   C3 R: 5’-CAGTGAAGATCCGATATAAG-3’
4. Chitinase 3-like-1
   Chil 1 F: 5’-CTGATCAAGGAACTGAATG-3’
   Chil 1 R: 5’-CGTAGGTCATGAGATTGATA-3’

Table S2. Histopathology scores of mice inoculated either with, [1] a high dose (3-4 x 10^7 CFU/5µL PBS) of *B. pertussis*, [2] three treatments of 10µL gentamicin (45µg) 8 hours apart and inoculated after 12 hours with 3-4 x 10^7 CFU/5µL PBS, [3] 5 µL PBS only, or [4] Antibiotic (gentamicin 45µ/10uL) only. Sev –severity; Dist - distribution

Date of Necropsy	Strain	Group	DPI	Mouse ID	Histo #	Nose Levels 1-4 Inflammation			Nose Levels 1-4 Hyaline globules			Nose Levels 1-4		Brain - olfactory bulb Inflammation		
						Sev	Dist		Sev	Dist		Biofilm	Mucus	Sev	Dist	
12/24/2018		1 (PBS + B. pertussis)	7	1	R19-630	3	3		0	0		yes	yes	2	2	
				2	R19-631	3	3		0	0		yes	yes	2	1	
				3	R19-632	3	3		0	0		yes	yes	2	1	
				4	R19-633	3	3		0	0		yes	yes	2	2	
		2 (Gentamicin + B. pertussis)	7	5	R19-634	3	3		0	0		yes	yes	2	1	
				6	R19-635	3	3		0	0		yes	yes	1	1	
				7	R19-636	3	3		0	0		yes	yes	1	1	
				8	R19-637	3	3		0	0		yes	yes	1	1	
		3 (PBS)	7	9	R19-638	0	0		0	0		yes	no	0	0	
				10	R19-639	0	0		0	0		yes	no	0	0	
		4 (Gentamicin)		11	R19-640	0	0		0	0		yes	no	0	0	
				12	R19-641	0	0		0	0		yes	no	0	0	

DPI = DAYS POST INFECTION

HISTOPATHOLOGIC SCORES WERE ASSIGNED AS GRADES 0 (NO SIGNIFICANT HISTOPATHOLOGICAL ALTERATIONS); 1 (MINIMAL); 2 (MILD); 3 (MODERATE);SPECIFIED
OR 4 (SEVERE) BASED ON AN INCREASING EXTENT AND/OR COMPLEXITY OF CHANGE , UNLESS OTHERWISE SPECIFIED

DISTRIBUTION SCORING KEY: 1 - FOCAL; 2 - MULTIFOCAL; 3 - DIFFUSE

SCORED BY/DATE: Uriel Blas-Machad0 DVM, PhD, DACVP 27Feb2019

Table S3. Differential gene expression in nasal cavity tissue from mice during infection with *Bordetella bronchiseptica.* Table depicts 11 genes exhibiting changes over time in expression levels (log2 fold change) over time in the nasal cavities of mice inoculated with B. bronchispetica. The analysis reveals induction of a strong innate immune response starting as early as 3-days postinfection lasting throughout the time course to day 21. mRNA transcripts of *B. pertussis Chil-1*, *Cd177*, *C3* and *muc* gene homolog were anlayzed in this study.

Gene	Symbol	Function	log2 (fold change) at day p.i.			
			3	7	14	21
Chitinase-like 1	*Chil1*	Th2 inflammatory response, antimicrobial	2.67	2.46	2.86	2.73
CD177	*Cd177*	Neutrophil degranulation	2.48	2.65	3.10	2.61
Complement Component C3	*C3*	Activation of the Complement system	1.67	1.30	2.00	1.93
Ceruloplasmin	*Cp*	Iron homeostasis	1.38	1.25	1.59	1.30
Regenerating islet-derived 3 gamma	*Reg3g*	Secretion of bactericidal C-type lectin	1.10	1.25	1.66	1.46
TNFα induced protein 2	*Tnfaip2*	Inflammation mediator	1.32	1.36	2.27	1.91
Interferon induced transmembrane protein	*Ifitm1*	IFN-induced antiviral protein	1.27	1.40	1.38	1.56
Mucin 1	*Muc1*	O-linked glycosylation of mucins	1.31	1.11	1.33	1.22
Mucin 4	*Muc4*	O-linked glycosylation of mucins	1.33	1.27	2.02	1.64
Mucin 5	*Muc5ac*	O-linked glycosylation of mucins	1.68	2.43	3.53	3.18
Polymeric immunogobulin receptor	*Pigr*	Immunoglobulin transcytosis	1.12	1.13	2.33	2.27
